# Reaction Mechanisms and Early-Stage Properties of Sustainable Calcium Carbide Residue-Granulated Blast Furnace Slag-Fly Ash Alkali-Activated Composites

**DOI:** 10.3390/ma19112382

**Published:** 2026-06-03

**Authors:** Haozhe Pan, Xingpei Yan, Stuart Thomas Wagland, Quan Liu

**Affiliations:** 1 Faculty of Materials and Civil Engineering, Jingjiang College, Jiangsu University, Zhenjiang 212028, China; hzpan@ujs.edu.cn; 2 Faculty of Engineering and Applied Sciences, Cranfield University, Bedfordshire MK43 0AL, UK; s.t.wagland@cranfield.ac.uk; 3 Faculty of Civil Engineering and Mechanics, Jiangsu University, Zhenjiang 212013, China; 2232423003@stmail.ujs.edu.cn

**Keywords:** early-strength materials, alkali-activated material, calcium carbide residue, hydration mechanism, environmentally friendly

## Abstract

**Highlights:**

- With a CCR content of 3%, an alkali content of 8% and a modulus of 1.4, the ternary CCR-GBFS-FA mortar achieves a compressive strength of 80.2 MPa on the 7th day.
- The addition of 3% CCR supplements Ca^2+^ and promotes the densification of the C-(N)-A-S-H gel; however, excessive use will result in the residual formation of CH crystals, thereby degrading the system.
- An activator modulus of 1.4 favors the polymerization reaction, whereas excessive alkali accelerates the formation of a gel layer, which in turn restricts the hydration process.
- Compared with conventional SAC, the low-carbon mortar developed in this study reduces carbon emissions by 64.6% and energy consumption by 48.6%, while maintaining high early-stage strength.

**Abstract:**

Infrastructure maintenance and emergency repairs require rapidly setting cementitious materials, yet conventional cement presents issues of high energy consumption and substantial CO_2_ emissions. Addressing this challenge, this research has developed a ternary alkali-activated cementitious material (CGFM) composed of calcium carbide residue (CCR), granulated blast furnace slag and fly ash. This study separately investigates the effects of CCR content (0–10%), alkali content (6–12%) and activator modulus (1.0–1.5) on workability and early mechanical strength. The hydration mechanism was examined through X-ray Diffraction (XRD), Fourier Transform Infrared (FTIR), Thermogravimetry-Derivative Thermogravimetry (TG-DTG) and Scanning Electron Microscopy-Energy Dispersive Spectroscopy (SEM-EDS) analysis, whilst life cycle assessment was employed to quantify the ecological impacts. Results indicated that a 3% CCR dosage significantly improved the gel structure, achieving a 7-day compressive strength of 69.8 MPa and a 37% increase in flexural strength. At a CCR dosage of 3%, alkali content of 8%, and modulus of 1.4, CGFM achieved a peak compressive strength of 80.2 MPa by the seventh day. This performance is attributable to its substantial gel content and high degree of polymerisation, which results in a dense structure. Life cycle assessment confirmed that compared to sulphoaluminate cement mortar, CGFM mortar reduced CO_2_ emissions by 64.6% and energy consumption by 48.6%.

## 1. Introduction

In projects such as emergency repairs at transport hubs, airport runway maintenance, and infrastructure construction, repair materials face exceptionally demanding time constraints. Specifically, these materials must achieve extremely high mechanical strength within a short period to restore service capability [1,2]. Although fast-hardening sulphoaluminate cement (SAC) and early-strength Portland cement have long supported such engineering demands [3,4], their production processes involve substantial energy consumption and CO_2_ emissions. According to statistics, the cement industry contributes approximately 6–13% of global CO_2_ emissions [5,6,7]. Given the current global imperative for carbon reduction and sustainable development, the construction industry faces significant pressure to identify low-carbon cementitious materials capable of replacing traditional highly energy-intensive cement [8]. Among these, alkali-activated materials prepared by activating aluminosilicate precursors such as fly ash and slag with alkali activators are regarded as one of the most promising alternatives due to their significantly low carbon footprint and excellent mechanical potential [9,10,11].

Under alkali-activated conditions, the high CaO, SiO_2_, and Al_2_O_3_ content in slag facilitates the rapid formation of a dense calcium aluminate silicate hydrate (C-A-S-H) gel, which in turn yields superior early mechanical strength [12]. However, pure slag-based alkali-activated systems commonly encounter bottlenecks such as excessively rapid setting and severe drying shrinkage, which severely restrict their large-scale pumping and construction in practical engineering applications [13,14]. To improve this situation, researchers typically introduce fly ash (FA) for blending. Due to their spherical morphology, FA particles act like ball bearings, which improves the flowability of the mortar. Moreover, owing to their relatively low reactivity, they effectively regulate the hardening rate of the system [15,16]. Research by Ge et al. [17] further revealed that the binary system of slag and FA can form a more stable three-dimensional skeletal structure than either component alone through the synergistic coexistence of calcium silicate hydrate (C-S-H) and C-A-S-H gel. However, existing binary systems remain highly dependent on commercially synthesised alkali activators (water glass and sodium hydroxide) to drive the reaction. These activators are not only costly, but their preparation process itself is highly energy-intensive and emits substantial pollutants. Statistics indicate that commercial activators account for about 40–80% of the total carbon footprint of geopolymer concrete [18]. To further reduce reliance on commercial alkali and achieve deep resource recovery of solid waste, establishing a ternary cementitious system incorporating highly alkaline waste materials has become an inevitable choice. Among numerous solid wastes, calcium carbide residue (CCR) has drawn significant attention due to its unique chemical properties. As a by-product of the acetylene industry, CCR is rich in Ca(OH)_2_ and exhibits extremely high alkalinity [19,20]. Using CCR as a high-alkalinity substitute for part of commercial activators and utilising its active calcium components in synergy with slag and FA may yield significant economic and ecological benefits.

To date, research into the application of CCR in alkali-activated systems utilising FA and slag has achieved some progress. Shi et al. [21] utilised red mud and CCR to synergistically activate slag and FA. They investigated the impact of different curing methods on the system’s strength, finding that the mortar achieved a maximum compressive strength of 21.9 MPa at the seventh day. Jiang et al. [22] also used CCR, red mud, slag, and FA to prepare a novel low-carbon cementitious material. Among paste specimens prepared with different mix proportions, the maximum compressive strength recorded at 3 days was 6 MPa. Research also found that during the hydration process, the Na^+^ in red mud was replaced by Ca^2+^ from CCR, transitioning from a stable compound state to a free state. This interaction with hydroxyl groups created a strongly basic environment, thereby accelerating the hydration reaction rate of the system. Guo et al. [23] utilised alkali residue and CCR to synergistically activate slag and FA. Research demonstrates that CCR incorporation not only increases the early hydration rate but also promotes the formation of crystalline products (C-S-H gel and hydrotalcite), resulting in a significant boost in early strength. The mortar achieved a compressive strength of 26.1 MPa at day 7. The influence of different curing regimes on the materials’ compressive strength within the same system was also explored by Guo et al. [24]. The findings indicated that curing at 75 °C for 12 h promoted early hydration reactions, with the 7-day pure paste compressive strength reaching 19.7 MPa. Li et al. [25] studied an alkali-activated material composed of CCR, slag, and FA. By testing different ratios of slag–FA mixtures and CCR dosages, the results revealed that the 7-day maximum compressive strength of the mortar was 17.1 MPa. The study confirmed the presence of hydration products, including C–S–H gel, C–A–S–H gel and hydrated talc-type compounds, which help to optimise the pore structure and enhance the strength of the material. Gu et al. [26] prepared a pure-slurry cementitious material with a 7-day compressive strength of approximately 21 MPa using CCR, slag, FA, and a small amount of desulphurisation gypsum. Similarly, Zhang et al. [27] investigated the mix proportions of alkali-activated cementitious materials from solid wastes using slag and FA as precursors, stimulated by the alkali activator CCR. The results demonstrated that when the CCR content ranged between 20% and 40%, the alkali-activated cement mortar exhibited optimal strength, with a compressive strength of 16.6 MPa on the third day.

Although CCR has been preliminarily demonstrated to activate silica–alumina precursors, hydrating to form cementitious materials possessing a certain strength, current research primarily focuses on utilising CCR alone or in combination with other alkaline solid wastes to activate silica–alumina precursors. The thus-obtained cementitious materials often exhibit limited strength, failing to meet practical engineering requirements. Zhu et al. [28] therefore adopted a dual-activation approach using CCR together with commercial activators to activate slag and FA. However, constrained by the low alkali content setting, the slurry sample’s 28-day compressive strength reached merely 21.4 MPa. Consequently, the synergistic effects in CCR–GBFS–FA cementitious systems (CGFM) remain insufficiently understood. In particular, the evolution of reaction products and the strength-development mechanisms under high-calcium and high-alkalinity conditions have yet to be fully clarified.

To address this gap, this study investigates the performance and mechanisms of CGFM under different alkaline environments. Its novelty lies in achieving high early strength through the coupled regulation of CCR content, alkali content and activator modulus, while clarifying reaction–product evolution and environmental benefits. Through single-factor experiments, the effects of CCR dosage (0–10%), alkali content (6–12%) and activator modulus (1.0–1.5) on the early-stage properties of this system were investigated in turn. At the micro level, the phase transitions of hydration products and the degree of chemical bond polymerisation were qualitatively analysed via XRD and FTIR. The reaction extent under different mixing ratios was quantitatively assessed using TG-DTG. SEM-EDS was employed to observe the morphological evolution of the gel phase and changes in key element ratios. This elucidated the early reaction mechanism of the system, thereby enhancing understanding of the strength-development mechanism of CCR-based ternary cementitious materials. Furthermore, this study employed a life cycle assessment (LCA) method to conduct a comparative analysis between CGFM and SAC mortar, quantitatively evaluating their ability in terms of low-carbon emission reduction. The results of this work not only establish a theoretical foundation for applying solid waste cementitious materials in early-strength engineering, but also furnish technical guidance and assurance for developing green construction materials.

## 2. Materials and Methods

### 2.1. Materials

**Figure 1:**
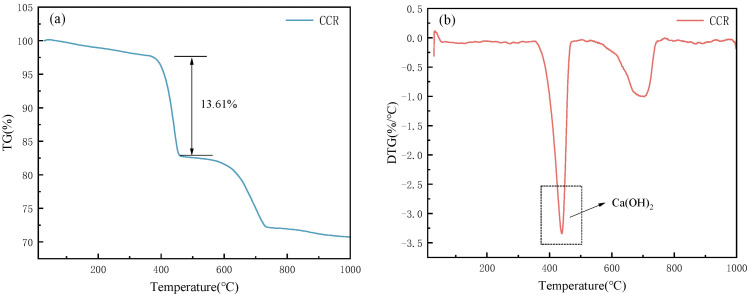
(**a**) TG of CCR, and (**b**) DTG of CCR.

**Figure 2:**
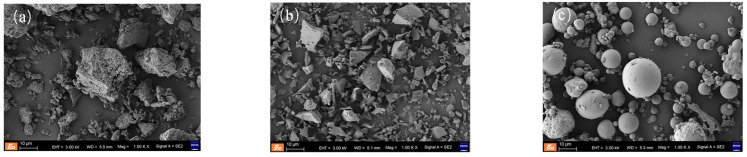
Micrograph of (**a**) CCR, (**b**) GBFS and (**c**) FA.

**Figure 3:**
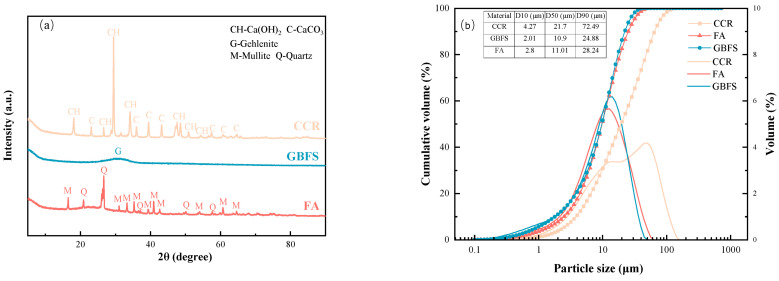
(**a**) Mineral composition of raw materials, and (**b**) particle size distribution of CCR, GBFS and FA.

In this study, three types of solid waste were used as cementitious components in the CGFM. The CCR was supplied by Jiuchang Building Materials Co., Ltd. in Zhucheng, China, whilst the GBFS and FA were both sourced from Bangneng Building Materials Co., Ltd. in Zhengzhou, China. The oxide-equivalent chemical compositions of the materials were determined by XRF analysis (Table 1). TG-DTG analysis was performed to further characterise the phases in CCR. As shown in Figure 1, CCR showed a distinct weight loss of approximately 13.61% at 400–500 °C, with a DTG peak at around 440 °C, which is attributed to the dehydroxylation of calcium hydroxide (CH). This confirms the presence of substantial CH in CCR. Figure 2 shows the microstructure of the raw material. CCR exhibits a relatively coarse surface with varied shapes. GBFS is comparatively smooth with distinct edges. FA, however, presents a more uniform spherical morphology. The mineral composition and particle size distribution of the materials were analysed using XRD and a laser particle size analyser (Figure 3). Figure 3a shows that the XRD pattern of GBFS exhibits distinct broad diffuse peaks and only weak crystalline peaks, indicating that GBFS consists primarily of a glassy amorphous structure. Water glass and sodium hydroxide (NaOH) serve as the supplementary alkali activators. The water glass used in the study was purchased from Jingcheng Chemical Co., Ltd., in Bengbu, China, with a modulus of 3.1 and a Na_2_O content of 8.41%. NaOH employed is a flake-like solid with a purity of 96%. The water employed was the local tap water from the laboratory, while the sand was standard sand conforming to the standard GB/T 17671-2021 (test method of cement mortar strength (ISO method)) [29].

### 2.2. Experimental Method

#### 2.2.1. Design Plan

Based on the previous study [28] and the reaction characteristics of alkali-activated materials, CCR content, alkali content, and activator modulus were selected as the main variables. Their effects on the early-stage performance of CGFM were investigated through staged single-factor experiments. The content of CCR is expressed as its mass ratio to all binders (CCR/B), including CCR, GBFS and FA. The alkali content is defined by the mass ratio of Na_2_O present in the activator to the binder materials (Na_2_O/B). Meanwhile, the modulus (Ms) refers to the molar ratio of SiO_2_ to Na_2_O within the activator. To prevent rapid setting caused by excessive CCR [25], CCR/B was set at 0%, 3%, 6% and 10% respectively. Owing to the high inertness of FA [30], the Na_2_O/B in this experiment was set at 6%, 8%, 10% and 12% to ensure the formation of a sufficiently alkaline environment for activating the precursor activity. Given that this study employed CCR as a synergistic alkaline activator, there was no need to use a low-modulus material to provide alkalinity. Consequently, we set the Ms to 1.0, 1.2, 1.4 and 1.5. In all mix designs, the mass ratio of GBFS to FA was fixed at 2:1, as higher GBFS content within the system is expected to yield higher early strength [31]. The water-to-binder ratio and sand-to-binder ratio were kept at 0.5 and 3, respectively. Drawing on previous research into alkali-activated materials [32] and employing a single-factor parameter control strategy, ten sets of mix designs were determined (Table 2). In the first stage, with Na_2_O/B and Ms fixed at 8% and 1.2, the effect of CCR/B was investigated at levels of 0%, 3%, 6% and 10%. In the second stage, based on the results of the first stage, CCR/B was fixed at 3% and Ms at 1.2, and the effect of Na_2_O/B was investigated at levels of 6%, 8%, 10% and 12%. In the third stage, with CCR/B and Na_2_O/B fixed at 3% and 8%, the effect of Ms was investigated at levels of 1.0, 1.2, 1.4 and 1.5.

#### 2.2.2. Sample Preparation

**Figure 4:**
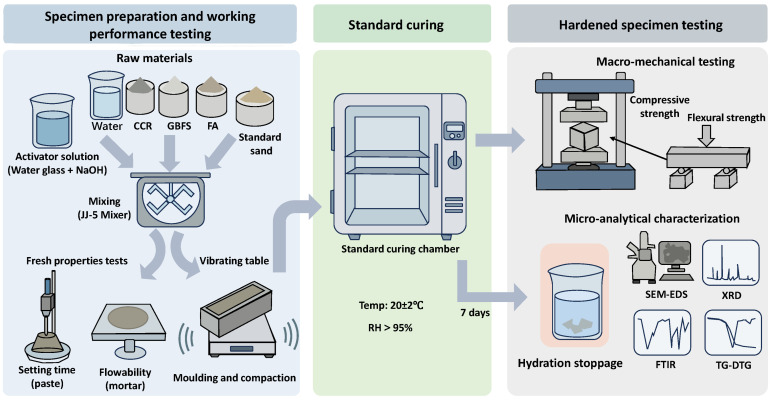
Preparation and testing procedure.

The preparation of specimens in this study broadly followed the procedure for preparing cement mortar. First, on the day prior to preparation, the activator solution (water glass and NaOH) was prepared according to the specified ratios. During preparation, CCR, GBFS and FA were pre-mixed in a mixer (JJ-5) for 20 s to achieve uniform distribution. Subsequently, the activator and water were added and stirred at low speed for 30 s. Then, the standard sand was poured in and stirred at low speed for another 30 s. The mixture was then left to rest for one minute before undergoing 60 s of high-speed stirring. Subsequently, the mixture was cast into a mould measuring 40 mm × 40 mm × 160 mm. The final mixture was placed on a vibrating table (ZS-15) and compacted to achieve uniform density. The moulded specimens were placed in a curing chamber maintained at 20 ± 2 °C with humidity exceeding 95%. Figure 4 illustrates the complete preparation process and corresponding tests.

#### 2.2.3. Mechanical Properties Testing

Mechanical testing was performed following the procedures specified in the standard GB/T 17671-2021 [29]. Specimens with dimensions of 40 mm × 40 mm × 160 mm were used for flexural and compressive strength tests. After flexural testing, the six fractured halves were subjected to compressive strength testing. Specimens cured for 3 and 7 days respectively were subjected to flexural and compressive strength testing on an electronic universal testing machine (DY-208JC). The loading rates applied during flexural and compressive strength tests were 50 N/s ± 10 N/s and 2400 N/s ± 200 N/s, respectively.

#### 2.2.4. Fresh Properties Testing

This research tested the two primary properties affecting the fresh properties of CGFM: setting time and flowability. The determination of setting time employed a paste specimen without sand via Vicat apparatus with reference to the standard GB/T 1346-2024 (test methods for water requirement of standard consistency, setting time and soundness of the Portland cement) [33]. The initial setting was recorded when the setting needle (diametr 1.03 ± 0.05 mm) was 4 ± 1 mm from the base plate. The specimen was then rotated 180 degrees and the setting needle replaced. The final setting was recorded when the final setting needle left no trace on the specimen’s surface. The flow test method was the jump table method according to the standard GB/T 2419-2005 (test method for fluidity of cement mortar) [34], employing a cement paste flow meter (NLD-3).

#### 2.2.5. Microscopic Testing Methods

To conduct an in-depth analysis of the macro-testing results, this study employed multiple micro-analysis methods for characterisation, including SEM ((Sigma 300, Zeiss, Oberkochen, Germany), EDS (Xplore 30, Oxford Instruments, Oxford, UK), XRD (Empyrean, PANalytical, Almelo, The Netherlands), FTIR (Alpha, Bruker, Karlsruhe, Germany), and TG-DTG (TG 209F1, Netzsch, Selb, Germany). All specimens tested were pre-treated by immersion in anhydrous ethanol to halt hydration, ensuring they accurately reflected their true state at the corresponding age. After hydration, the samples were dried at 40 °C. Fresh fractured surfaces were selected and gold-coated before SEM observation. The secondary electron detector of the SEM was set to an acceleration voltage of 3 kilovolts. The XRD analysis was performed using Cu Kα radiation with a wavelength of 1.541874 Å. Measurements were taken in the range from 5° to 90°, at a scanning speed of 5°/min and in 0.02° steps, under conditions of 40 kV and 40 mA. The TG heating range was from room temperature to 1000 °C, with a heating rate of 10 °C/min. The wavelength range for FTIR testing ranged from 4000 cm^−1^ to 400 cm^−1^.

#### 2.2.6. Data Processing

To limit experimental error, the flow test results were determined by measuring the average diameter of the specimen in two perpendicular directions after vibration. For mechanical properties, flexural strength was averaged from three prism specimens, and compressive strength was averaged from six readings obtained from the same three cubic specimens. The standard deviation is displayed as error bars on the mechanical properties bar chart.

## 3. Results and Discussion

### 3.1. Fresh Properties

**Figure 5:**
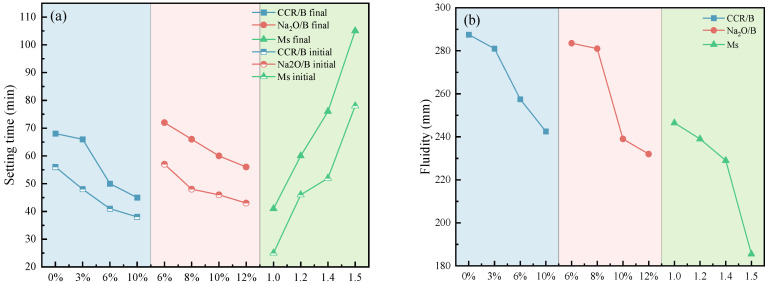
(**a**) Setting time of the slurry, and (**b**) flowability of the mortar.

To evaluate the workability of CGFM, this study tested the setting time and fluidity under different mix proportions. The specific test results are summarised in Table 3, with the trend of change presented visually in Figure 5. The results indicate that the setting time and fluidity of this system exhibited high sensitivity to the CCR/B, Na_2_O/B and Ms. As shown in Figure 5a, the addition of CCR significantly accelerated the setting process of the paste. As the CCR dosage increased from 0% to 10%, the initial setting time decreased from 56 min to 38 min, while the final setting time correspondingly decreased from 68 min to 45 min. This trend is consistent with the finding of Gu et al. [35], which indicated that calcium-rich additives could effectively shorten the reaction time of alkali-activated materials. CCR contains highly crystalline CH, which rapidly dissolves upon contact with the alkali activator, releasing high concentrations of Ca^2+^ and OH^−^. These ions act as nucleation sites, promoting the rapid precipitation and growth of C-(A)-S-H gel [36]. Similar to the CCR pattern, the setting time gradually shortened as the Na_2_O/B increased from 6% to 12% (initial setting time decreased from 57 min to 43 min). This observation agrees with the results reported by Du et al. [37], in which a higher Na_2_O content leads to an increase in solution pH, which in turn greatly accelerates the dissolution of the silicoaluminate precursor and consequently speeds up the polycondensation reaction of the material. As shown in Figure 5a, the setting time significantly extended when Ms increased from 1.0 to 1.5 (initial setting time increased from 25 min to 78 min). This result is consistent with the finding of Li et al. [38], who found that an increase in the viscosity of high-modulus solutions reduced their diffusion rate within the slurry, thereby prolonging the gelation time of the system.

Figure 5b illustrates the effects of CCR/B, Na_2_O/B, and Ms on the fluidity of the mortar. When the CCR/B increased, the mortar’s fluidity exhibited a monotonically decreasing trend. When the CCR/B reached 10%, the fluidity decreased from 287.5 mm to 242.5 mm. This is likely due to CCR particles typically having a loose, porous structure and a high specific surface area, physically adsorbing substantial quantities of free water within the system [39]. Similarly, as Na_2_O/B increased, the fluidity decreased from 283.5 mm to 228 mm. The deterioration in fluidity at high alkali content may be primarily attributable to the increased viscosity of the alkali activator solution itself. High concentrations of activator form a highly viscous medium, significantly increasing the plastic viscosity of the mortar [40]. It is noteworthy that when the Ms increased from 1.0 to 1.4, the fluidity decreased only moderately by 7%, whereas when the Ms reached 1.5, the fluidity dropped sharply by approximately 19% (Figure 5b). This may be attributed to the high viscosity of high-modulus activators, which significantly increases the internal friction throughout the slurry, thereby reducing its flowability [41].

### 3.2. Mechanical Properties

**Figure 6:**
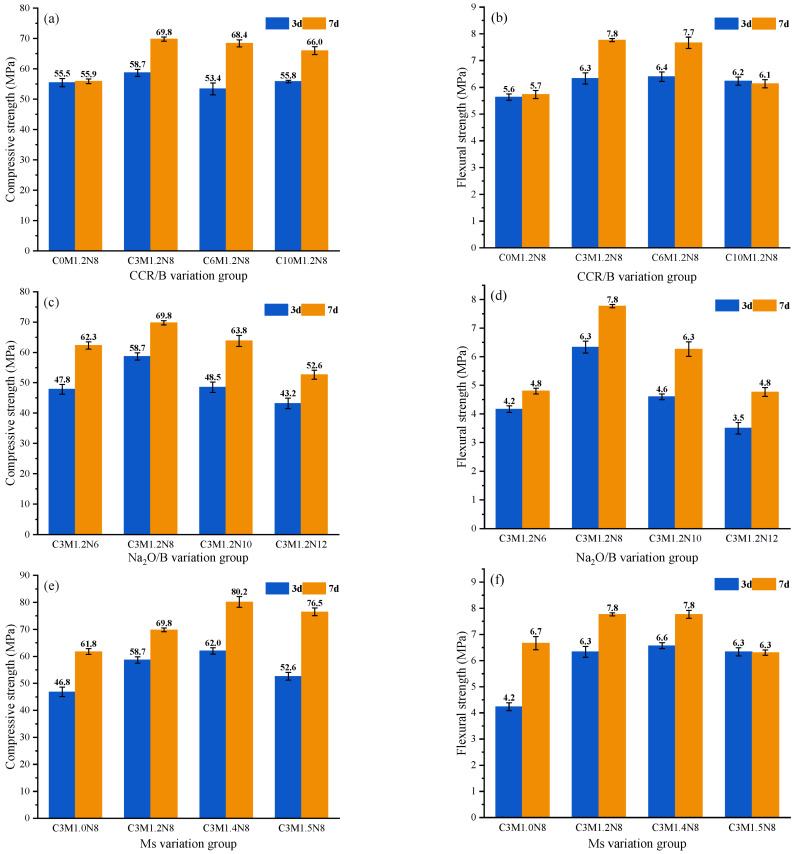
(**a**) Effect of CCR on compressive strength; (**b**) effect of CCR on flexural strength; (**c**) effect of Na_2_O/B on compressive strength; (**d**) effect of Na_2_O/B on flexural strength; (**e**) effect of Ms on compressive strength; (**f**) effect of Ms on flexural strength.

This study tested the compressive and flexural strength of CGFM at 3- and 7-day age to comprehensively evaluate their mechanical properties. Detailed data are summarised in Table 3, with the specific trends of different mix design parameters affecting compressive and flexural strength presented in Figure 6a–f. Figure 6a illustrates that incorporating 3% CCR increased the 7-day compressive strength from 55.9 MPa in the C0M1.2N8 to 69.8 MPa, while overcoming the strength stalling observed in the C0M1.2N8 between the 3- and 7-day age. The flexural strength exhibited a more pronounced enhancement effect (Figure 6b). The C0M1.2N8 mixture achieved a 7-day flexural strength of only 5.7 MPa, while that of C3M1.2N8 reached 7.8 MPa, representing a 37% improvement. However, when CCR/B became excessively high (10%), the 7-day flexural strength declined to 6.1 MPa. This trend aligns with the finding of Yang et al. [42], which indicates that an appropriate amount of calcium sources can optimise gel structure and enhance matrix toughness. However, the presence of excessive CH may create weak zones prone to stress concentration, leading to a decline in strength.

As shown in Figure 6c,d, as Na_2_O/B increased, both compressive and flexural strength displayed a trend of initially rising before declining, reaching their peak at a Na_2_O/B of 8% (C3M1.2N8). As Na_2_O/B increases, more silicoaluminate precursors dissolve and participate in the polymerisation reaction, forming a denser and more uniform gel network structure, thereby enhancing the material’s mechanical properties [32]. However, excessively high alkalinity may lead to elevated ion concentrations within the solution, which in turn inhibits the further dissolution and depolymerisation of silicates and aluminates. This results in the formation of an uneven gel structure, thereby diminishing the system’s strength [41]. It also should be noted that the compressive-to-flexural strength ratio for C3M1.2N12 was approximately 11.0, significantly higher than the 8.9 observed for C3M1.2N8. This phenomenon may suggest that the cementitious system formed in a high-alkali environment tends to exhibit considerable brittleness.

With increasing Ms of the activator, the mechanical strength of the cementitious system also presented a similar trend to the change in Na_2_O/B (Figure 6e,f). The group with a Ms of 1.4 (C3M1.4N8) achieved the highest compressive strength (80.2 MPa) and flexural strength (7.8 MPa), demonstrating outstanding mechanical properties. This is similar to the finding of Xu et al. [43] and may be because the increased Ms raised the soluble SiO_2_ content within the system, providing raw material for the formation of gels, thereby significantly enhancing the strength [44]. Nevertheless, under constant Na_2_O/B, an increase in the modulus leads to a reduced concentration of free OH^−^ ions in the solution, which may suggest a decline in the overall extent of the reaction [45]. Consequently, when the Ms increased to 1.5, a decline in strength occurred.

Related reports on CCR-based alkali-activated systems (involving slag, fly ash, and red) have shown moderate early strength, with 7-day compressive strengths commonly lower than 35 MPa [46,47]. By contrast, the optimised C3M1.4N8 mixture in this study achieved a 7-day compressive strength of 80.2 MPa, demonstrating the effectiveness of the coupled regulation of CCR/B, Na_2_O/B and Ms for developing high-early-strength materials.

### 3.3. Phase Composition Analysis

To analyse the early hydration characteristics of CGFM, the samples were characterised using XRD, FTIR, TG, and SEM-EDS.

#### 3.3.1. XRD Analysis

**Figure 7:**
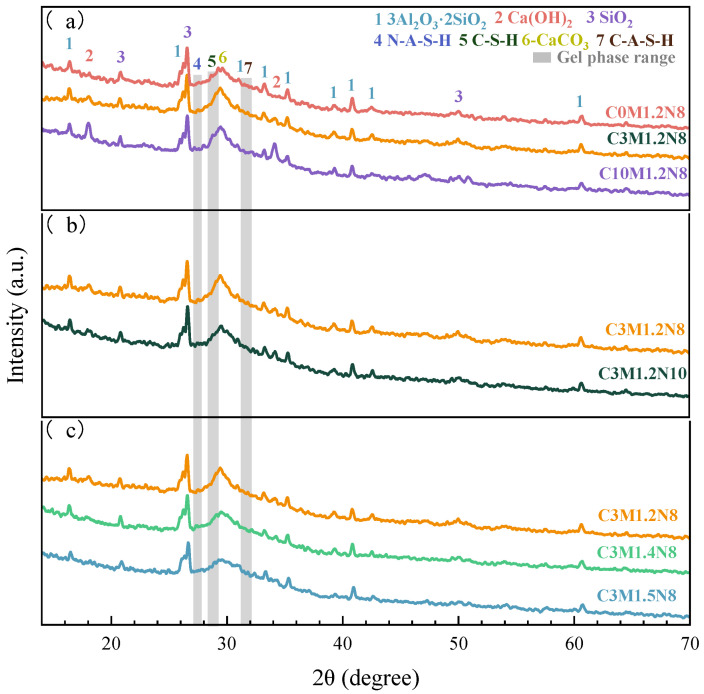
7-day XRD results for specimens with different mixing ratios: (**a**) different CCR/B; (**b**) different Na2O/B; (**c**) different Ms.

This study conducted XRD analysis on the paste samples at the 7-day age. The XRD diffraction patterns for all samples are shown in Figure 7. The patterns exhibited partially sharp crystalline peaks alongside a series of broadened amorphous diffuse peaks. The crystalline phases primarily comprised 3Al_2_O_3_∙2SiO_2_, CH, SiO_2_, and CaCO_3_. Through analysis using Jade software 6.0, and in conjunction with the relevant literature [48,49,50], the broad diffraction response in the 27–32° range may be associated with the formation of amorphous calcium/sodium aluminosilicate gel-like products (C-(N)-A-S-H).

As can be observed from Figure 7a, the gel peak in the C3M1.2N8 was enhanced compared to that in the C0M1.2N8. This result may suggest that small quantities of CCR could effectively supplement calcium sources within the system, synergising with slag to promote the formation of the gel network. When CCR/B continued to increase to 10%, distinct CH crystalline diffraction peaks appeared in the pattern, although the gel peak retained its high intensity. This observation is consistent with earlier mechanical test outcomes, suggesting that an appropriate dosage of CCR facilitates the development of an amorphous C-(N)-A-S-H gel. In contrast, excess CCR hinders the complete incorporation of Ca^2+^ into the gel network, resulting in its retention as unreacted CH crystals. Figure 7b shows that as the Na_2_O/B increased from 8% to 10%, the overall peak intensity of the gel phase did not show a significant increase. This phenomenon is consistent with the finding of Dang et al. [51], who observed that at lower Ms values, an excessively high alkali content (10% Na_2_O) may lead to an overly rapid reaction, resulting in the formation of a loose product coating. This also accounts for the observed decrease in strength for the C3M1.2N10 sample. Comparing the diffraction curves of different Ms in Figure 7c, it is evident that with increasing Ms, the peak intensities of CH (around 18°) gradually diminished. This may suggest that as the silicon source within the system increases, it consumes excess CH. As for the peak strength of the system occurring at a Ms of 1.4 and the subsequent decline in strength at a Ms of 1.5, this requires further analysis in conjunction with subsequent microscopic testing.

#### 3.3.2. FTIR Analysis

**Figure 8:**
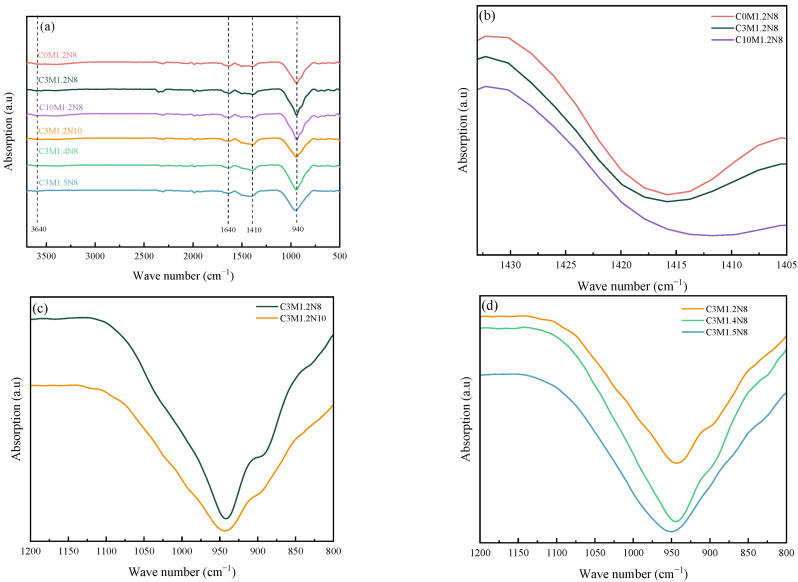
FTIR spectra of paste samples: (**a**) all samples; (**b**) different CCR/B; (**c**) different Na2O/B; (**d**) different Ms.

To further elucidate the macroscopic phenomena, FTIR analysis was performed on paste samples aged for 7 days, verifying the degree of polymerisation within the gel structure from the perspective of molecular bond energy. As can be seen from Figure 8a, the principal characteristic absorption bands in the spectrum fall into the following categories. The frequencies around 3640 cm^−1^ and 1640 cm^−1^ primarily corresponded to the stretching and bending vibrations of the O-H bond in crystalline water. The absorption peak within the 1410–1450 cm^−1^ range corresponded to the asymmetric stretching vibration of the O–C–O bond, indicating the presence of carbonate. The peak shift at 940–1050 cm^−1^ originated from the asymmetric stretching of Si–O–T bonds (T = Si, Al). This peak displacement serves as a key criterion for determining the polymerisation degree and chemical component of gel [52,53].

Figure 8a shows that as the CCR content increased from 0% to 3%, the Si-O-T absorption peak correspondingly intensified. This result reflects that CCR provides sufficient Ca^2+^ and OH^−^, promoting the depolymerisation and repolymerisation of fly ash and slag glass phases. When the CCR content was further increased to 10%, although its main peak absorption intensity approached that of C3M1.2N8, the significant residual CH observed in the XRD analysis indicated that the excess CCR failed to fully participate in the pozzolanic reaction. Thus, the macroscopic strength (66.0 MPa) was lower than that of C3M1.2N8 (69.8 MPa). Furthermore, in the 1410–1450 cm^−1^ region, the carbonate absorption peak of C10M1.2N8 shifted towards lower wavenumbers to approximately 1411 cm^−1^ and increased in intensity (Figure 8b). This feature agrees with the XRD findings, in which sharp peaks corresponding to calcite and CH were detected for the C10M1.2N8 sample.

When the Na_2_O/B increased from 8% to 10%, the peak intensity of Si-O-T correspondingly weakened (Figure 8c). The reduction in absorption intensity indicates a decrease in the effective gel formation, which aligns closely with the macroscopic trend of compressive strength declining from 69.8 MPa to 63.8 MPa. Figure 8d reveals that higher Ms values shifted the Si–O–T peak to higher wavenumbers, indicating increased gel polymerisation [53]. Meanwhile, C3M1.4N8 also maintained a high absorption peak intensity, suggesting the system achieved synergistic effects between high polymerisation degree and high gel yield. This may also account for its attainment of the highest mechanical strength. Although the peak position of C3M1.5N8 shifted further to the highest point (around 951.41 cm^−1^), indicating the highest degree of polymerisation, the peak intensity diminished, revealing a decrease in total gel content. Consequently, the overall intensity exhibited a decline.

#### 3.3.3. TG/DTG Analysis

**Figure 9:**
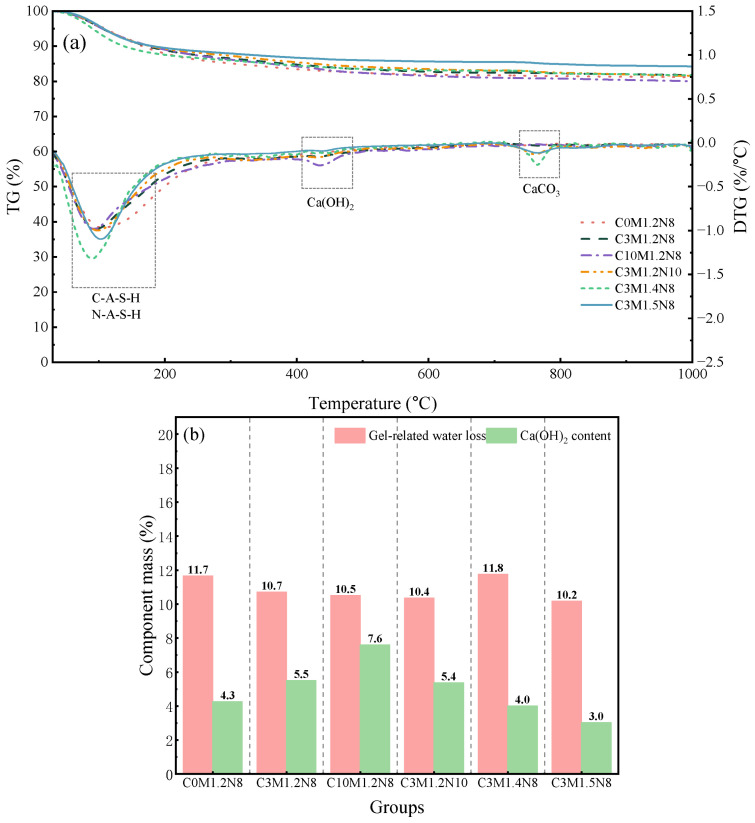
TG-DTG curves and substance content at different ratios: (**a**) TG-DTG curves; (**b**) gel water loss, CH content and total loss.

FTIR analysis suggests that variations in gel content across different groups may account for differences in mechanical performance. Consequently, this study conducted TG-DTG on 7-day paste samples across a temperature range of 0–1000 °C to quantitatively analyse hydration products. For the convenience of analysis, the content of CH was determined based on the dihydroxylation weight loss data, as shown in Equation (1). As can been seen in Figure 9a, the weight loss of all samples essentially exhibited three distinct stages. Mass loss at 50–200 °C is mainly attributed to the desorption of physically adsorbed water and chemically bound interlayer water from C-(N)-A-S-H gels [21,25,54]. Under identical processing and testing conditions, this mass loss can only serve as a relative indicator of gel-like hydration product formation. The mass losses at 400–500 °C and 600–750 °C are assigned to the decomposition of CH and CaCO_3_, respectively.

Figure 9b demonstrates that as the CCR/B increased from 0% to 10%, the weight loss of CH significantly increased. The residual CH content of C10M1.2N8 (7.6%) indicates that excessive CCR failed to fully participate in the pozzolanic reaction. This is highly consistent with the increasing trend in the intensity of the CH diffraction peak in XRD. It is worth noting that although the gel-related water loss of C3M1.2N8 (10.70%) was slightly lower than that of C0M1.2N8 (11.66%), it exhibited higher compressive strength (69.8 MPa). Combined with the FTIR results, this may be because, while the weight loss was comparable, the C3M1.2N8 system exhibited a higher degree of polymerisation, resulting in a denser matrix.

At a Ms of 1.2, increasing the Na_2_O/B from 8% to 10% resulted in a decrease in gel-related water loss from 10.70% to 10.35%. This data quantitatively corroborates the earlier FTIR interpretation regarding reduced gel content. C3M1.4N8 displayed the highest gel water loss (11.76%) across all the groups. As a key indicator reflecting the total amount of gelation products, this result explains why it achieved the highest compressive strength of 80.2 MPa. In contrast, the gel dehydration loss of C3M1.5N8 decreased significantly to 10.17%, and its CH content was also the lowest in the entire group (3.02%). This indicates that at the high Ms (1.5), despite FTIR indicating a higher degree of polymerisation, the overall reaction yield was constrained by insufficient alkalinity. This explanation is consistent with the findings of relevant study [55]. Consequently, its compressive strength (76.5 MPa) was inferior to that of the 1.4 Ms group.

CH content = CH mass loss × Molar mass of CH/Molar mass of H_2_O (1)

#### 3.3.4. SEM-EDS Analysis

**Figure 10:**
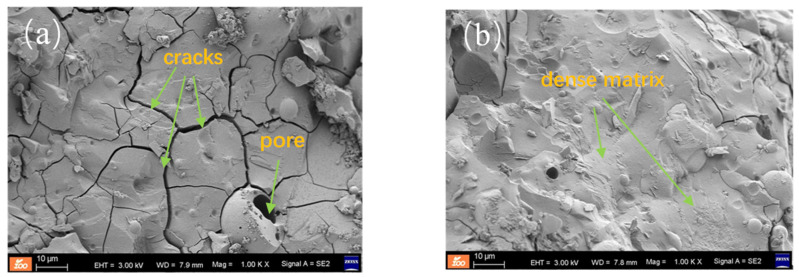

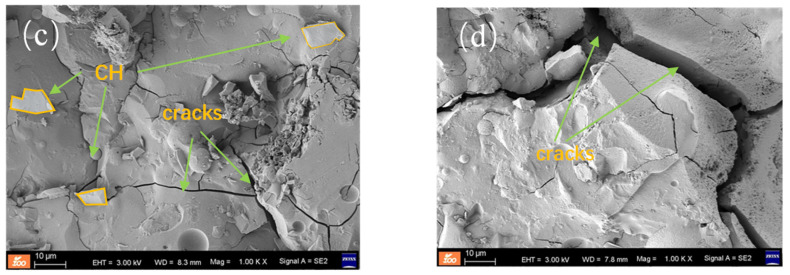
Microstructural morphology at different mixing ratios: (**a**) C0M1.2N8; (**b**) C3M1.2N8; (**c**) C10M1.2N8; (**d**) C3M1.2N10.

To visually observe the microstructure of the hydration products and quantitatively analyse the chemical composition of the gel phase, this study utilised SEM and EDS surface scan techniques on 7-day paste specimens. Figure 10a reveals that the microstructure of C0M1.2N8 exhibited numerous microcracks and unreacted pores, indicating incomplete hydration and a poorly compacted gel structure. This corresponds to its lowest 7-day compressive strength of 55.9 MPa. C3M1.2N8 demonstrated a more continuous and denser matrix structure (Figure 10b). EDS analysis showed a Ca/Si ratio of 1.27 and a relatively low Na/Si ratio of 0.37, suggesting that moderate CCR addition provides both alkalinity and Ca^2+^, thereby favouring the formation of calcium-rich C-(N)-A-S-H gels [56]. Figure 10c shows the presence of distinct CH crystals in C10M1.2N8. Combined with XRD and TG analysis (CH content reaching 7.6%), these findings indicate that excess CCR leads to the enrichment of CH crystals, thereby weakening inter-gel bonding and explaining the decline in strength.

The gel matrix of C3M1.2N10 displayed pronounced cracking (Figure 10d). Combined with EDS results, its Ca/Si ratio (0.81) was significantly lower than that of C3M1.2N8 (1.27), indicating impeded Ca^2+^ incorporation into the gel, thereby inhibiting the formation of C-(A)-S-H. Through combined FTIR and TG analysis, it may be inferred that excessively high alkalinity likely formed a dense product layer enveloping the raw material particles during the initial reaction stage. This resulted in reaction inhibition, reduced gel yield, and structural discontinuities. Accordingly, the 7-day compressive strength receded to 63.8 MPa.

**Figure 11:**
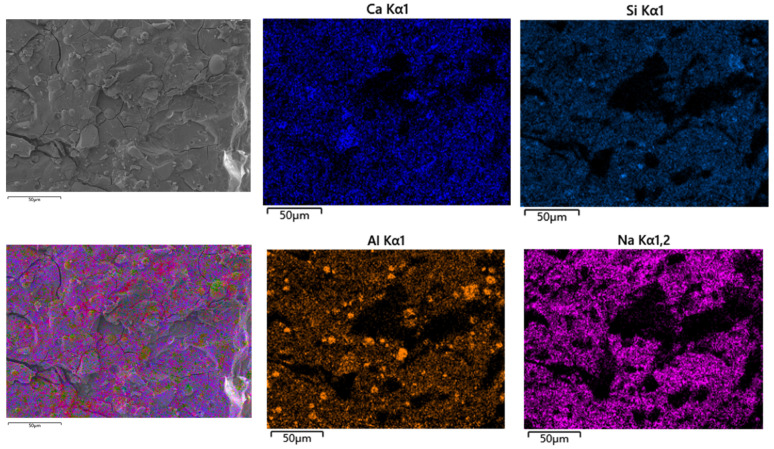
Microstructure and EDS element distribution in the C3M1.4N8 sample.

As can be seen from Figure 11, C3M1.4N8 presented a dense, homogeneous morphology. EDS surface scans further validated the highly overlapping uniform distribution of Ca, Si, Al, and Na across the micrometre scale. As shown in Table 4, C3M1.4N8 showed a moderate Ca/Si ratio (0.78), a stable Al/Si ratio (0.39), and a relatively high Na/Si ratio (0.64), suggesting sustained Al incorporation into the aluminosilicate framework and an enhanced role of Na in charge balancing. This chemically balanced C-(N)-A-S-H gel network may explain its optimal compressive strength. However, increasing Ms to 1.5 reduced the Al/Si ratio to 0.35, indicating limited Al incorporation, fewer effective gel-like products, and lower strength.

### 3.4. Hydration Mechanism

**Figure 12:**
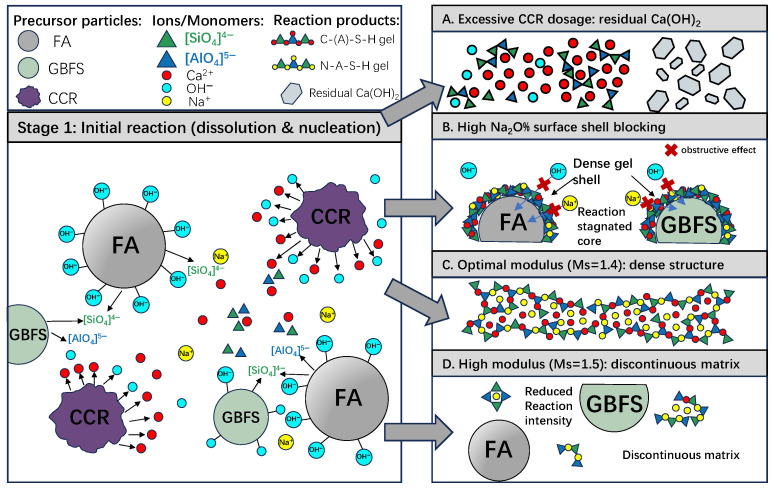
Schematic diagram of CGFM hydration mechanism.

Based on the macroscopic performance and microstructure analysis, this study proposes a reaction mechanism explanation for CGFM under different excitation environments (Figure 12). In the initial stage of the reaction, high concentrations of OH^−^ rapidly attack the vitreous skeleton of GBFS and FA. The addition of CCR provides additional Ca^2+^ and OH^−^, with the dissolved Ca^2+^ acting as a nucleating agent, accelerating the condensation polymerisation of [SiO_4_]^4−^ and [AlO_4_]^5−^ monomers [57]. However, excessive CCR prevents Ca^2+^ from fully entering the gel network, causing it to remain as residual CH instead. When the Na_2_O/B increases, the high pH value causes the precursor surface to dissolve too quickly, rapidly precipitating a dense gel layer on the particle surface. The gel layer blocks the channels for water to penetrate inward, causing the internal reaction to stagnate [58]. The Ms further regulates the Ca–Na–Al–Si balance in the binding products. At low Ms values, a calcium-rich gel-like matrix is favoured. As the Ms increases, the increased soluble silicate content promotes aluminosilicate polymerisation, accompanied by a lower Ca/Si ratio and higher Na/Si ratio, suggesting a transition toward a mixed C-(N)-A-S-H matrix [59]. This balance appears to be optimised at an Ms of 1.4, leading to a dense and continuous binding matrix. However, when the concentration of active silicate ions becomes excessively high, insufficient OH^−^ concentration may weaken the erosive force on the surfaces of FA and GBFS particles during the initial reaction phase, thereby reducing reaction intensity. Even if gel polymerisation degree increases, the overall decrease in total gel yield will still compromise the continuity of the matrix.

### 3.5. Environmental Impact Assessment

To quantify the ecological benefits of CGFM, this section systematically assesses the carbon footprint of CGFM mortar based on the life cycle assessment (LCA) method [60,61].

#### 3.5.1. Parameters and System Boundaries

Based on measured data from a standard prism specimen (volume 256,000 mm^3^, mass 486 g), the density of the CGFM mortar is calculated to be 1898 kg/m^3^. A 42.5-grade SAC mortar is selected for comparative analysis. The mixture ratio for standard 42.5-grade SAC mortar, as defined by standard GB/T 20472-2006 (sulphoaluminate cement) [62] and GB/T 17671-2021 [29], consists of cement, sand, and water in a ratio of 1:3:0.5, respectively. The corresponding density is approximately 2636 kg/m^3^, and the standard compressive strength after 3 days is 42.5 MPa. The embodied carbon emissions (EC) and embodied energy consumption (EE) values for raw materials are shown in Table 5. The functional unit of the LCA was defined as 1 m^3^ of mortar. A cradle-to-gate system boundary was adopted, including transportation, drying, calcination, and sieving. It should be noted that this paper did not conduct a comprehensive sensitivity analysis. The results obtained are dependent on the functional units, system boundaries and inventory assumptions specified in this paper.

#### 3.5.2. Carbon Emissions and Energy Consumption Analysis

**Figure 13:**
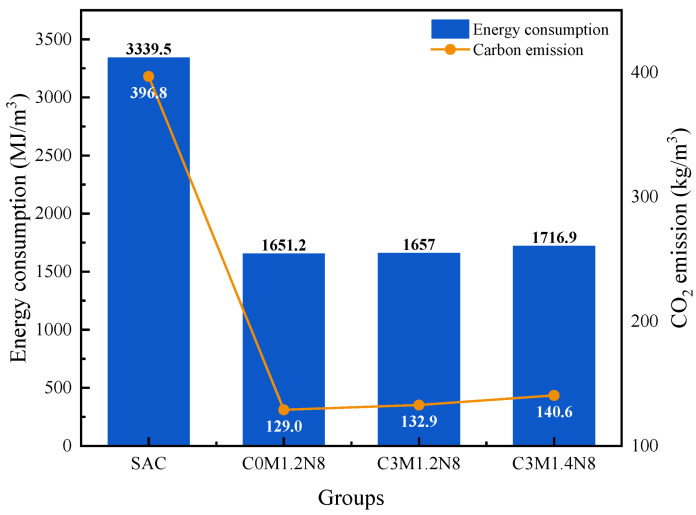
Carbon emissions and energy consumption by different groups.

Using the mix design parameters in Table 2 and the information in Table 5, the carbon emissions and energy consumption for different mortar groups can be calculated. The specific calculation method is shown in Equations (2) and (3). As shown in Figure 13, the SAC group exhibited total carbon emissions as high as 396.8 kg CO_2_/m^3^ and energy consumption of 3339.5 MJ/m^3^, primarily attributable to fuel consumption during carbonate decomposition and cement clinker production processes. In comparison, The CGFM group’s carbon emissions and energy consumption are both significantly lower than those of the SAC. When a small amount of CCR (3%) was incorporated for synergistic activation, both carbon emissions and energy consumption decreased to 132.9 kg/m^3^ and 1657 MJ/m^3^ respectively. Though the carbon emissions and energy consumption of C3M1.4N8 increased with rising activator modulus (140.6 kg/m^3^ and 1716.9 MJ/m^3^, respectively), it still achieved a 64.6% reduction in carbon emissions and a 48.6% decrease in energy consumption compared to SAC. Commercial activators are the dominant contributors to the environmental impact of the CGFM system. In the optimal mix C3M1.4N8, water glass and NaOH consumptions are estimated at 163.4 and 22.4 kg/m^3^, respectively, jointly accounting for 63.9% of its total carbon emissions.

Total CO_2_ = Σ Quantity of each material × EC (2)

Total energy consumption = Σ Quantity of each material × EE(3)

#### 3.5.3. Carbon and Energy Intensity Assessment

To comprehensively evaluate environmental performance relative to mechanical gain, this study introduced the carbon intensity (CI) and energy intensity (EI), defined as CO_2_ emissions per unit compressive strength (kgCO_2_/MPa) and the energy consumption per unit of compressive strength (MJ/MPa). The calculation method is shown in the following equation:

CI = Total CO_2_/Compressive strength (4)

EI = Total Energy/Compressive strength(5)

As shown in Table 6, C3M1.4N8 exhibited highly competitive carbon and energy intensity, with a value as low as 2.27 kgCO_2_/MPa and 27.7 MJ/MPa. Compared to SAC mortar (9.34 kgCO_2_/MPa and 78.6 MJ/MPa), related intensity was improved by 4.1 times and 2.8 times respectively. This demonstrates that the material can provide superior mechanical load-bearing capacity at extremely low environmental cost. Therefore, the above environmental assessment results strongly confirm the ecological superiority of CGFM mortar. By converting CCR into functional cementitious components, this system provides a scalable solution for the construction industry to simultaneously address the dual challenges of solid waste disposal and carbon neutrality.

## 4. Conclusions

This study successfully developed a green ternary alkali-activated cementitious material (CGFM) composed of CCR, GBFS and FA and systematically analysed its macroscopic properties and microscopic mechanisms. By systematically analysing their macroscopic properties and demonstrating the synergistic effects of CCR during the densification of the gel network, this study provided a theoretical basis for the application of solid waste-based cementitious materials in early-strength engineering. The main conclusions are as follows:

(1) Increasing the proportion of CCR accelerated the setting time of the mixture and reduced its workability. Raising CCR from 0% to 10% reduced the initial setting time from 56 to 38 min and substantially impaired the flowability. This is because the rapid dissolution of CH in CCR provides a large number of nucleation sites, accelerating the precipitation of the C-(N)-A-S-H gel, whilst its loose, porous structure physically adsorbs a significant amount of free water.
(2) An appropriate CCR content improved strength, but excessive addition impaired performance. The incorporation of 3% CCR increased the 7-day compressive strength from 55.9 MPa to 69.8 MPa and improved the flexural strength by approximately 37%. This is attributed to the fact that an appropriate amount of CCR supplements the calcium source in the system, synergising with the slag to promote the formation of a high-density C-(N)-A-S-H gel network. However, excessive incorporation (10%) leads to an accumulation of residual CH crystals, creating stress-concentration zones that weaken the structure and consequently reduce its strength.
(3) Under conditions of 8% Na_2_O/B and a Ms of 1.4, the CGFM achieved a maximum 7-day compressive strength of 80.2 MPa. Microstructural analysis indicates that this mix design achieved a synergy between high polymerisation degree and maximum gel yield (11.76% water loss), resulting in a continuous and dense matrix structure.
(4) When the Na_2_O/B (12%) or Ms (1.5) was too high, there was a marked decline in mechanical properties. This is primarily because extremely high alkalinity causes early reaction products to rapidly coat unreacted particles, resulting in an inhibitory effect. Conversely, an excessively high modulus reduces the erosive force on the aluminosilicate framework due to insufficient OH^−^ concentration, leading to a decrease in total gel yield.
(5) LCA analysis results indicated that the optimised CGFM (C3M1.4N8) mortar reduced carbon emissions by 64.6% and energy consumption by 48.6% compared to sulphoaluminate cement mortar. Its carbon and energy intensity was improved by 4.1 times and 2.8 times respectively, providing a sustainable solution for industrial solid waste utilisation.
(6) This study has demonstrated that CGFM possesses potential as an early-strength engineering material. However, it has also revealed a synergistic competitive mechanism between the activator modulus and alkali content, necessitating the identification of an optimal equilibrium point. This requires further investigation in subsequent research.

## Figures and Tables

**Table 1:** Oxide-equivalent chemical composition of cementitious components (%).

Materials	CaO	SiO_2_	Al_2_O_3_	SO_3_	Fe_2_O_3_	MgO	Na_2_O	K_2_O	TiO_2_	LOI
CCR	91.28	3.93	2.19	1.44	0.46	0.27	0.16	0.06	0.06	-
FA	4.33	52.92	30.39	0.95	5.37	0.99	0.66	2.40	1.03	-
GGBS	41.86	29.14	15.88	2.34	0.38	7.85	0.44	0.44	0.80	-

**Table 2:** Mixing ratio parameters.

Groups	Varied Factor	CCR/B%	GBFS:FA	GBFS/B%	FA/B%	Ms	Na_2_O/B%	W/B	S/B
C0M1.2N8	CCR/B	0	2:1	66.7	33.3	1.2	8	0.5	3
C3M1.2N8	Shared reference	3	2:1	64.7	32.3	1.2	8	0.5	3
C6M1.2N8	CCR/B	6	2:1	62.7	31.3	1.2	8	0.5	3
C10M1.2N8	CCR/B	10	2:1	60	30	1.2	8	0.5	3
C3M1.2N6	Na_2_O/B	3	2:1	64.7	32.3	1.2	6	0.5	3
C3M1.2N10	Na_2_O/B	3	2:1	64.7	32.3	1.2	10	0.5	3
C3M1.2N12	Na_2_O/B	3	2:1	64.7	32.3	1.2	12	0.5	3
C3M1.0N8	Ms	3	2:1	64.7	32.3	1.0	8	0.5	3
C3M1.4N8	Ms	3	2:1	64.7	32.3	1.4	8	0.5	3
C3M1.5N8	Ms	3	2:1	64.7	32.3	1.5	8	0.5	3

Note: All percentages are expressed by mass. C, M and N in the group name represent CCR/B, Ms and Na_2_O/B, respectively.

**Table 3:** Macroscopic test results for CGFM.

Groups	Initial/min	Final/min	Fluidity/mm	3d FS/MPa	7d FS/MPa	3d CS/MPa	7d CS/MPa
C0M1.2N8	56	68	287.5	5.6	5.7	55.5	55.9
C3M1.2N8	48	66	283.5	6.3	7.8	58.7	69.8
C6M1.2N8	41	50	257.5	6.4	7.7	53.4	68.4
C10M1.2N8	38	45	242.5	6.2	6.1	55.8	66
C3M1.2N6	57	72	281	4.2	4.8	47.8	62.3
C3M1.2N10	46	60	239	4.6	6.3	48.5	63.8
C3M1.2N12	43	56	232	3.5	4.8	43.2	52.6
C3M1.0N8	25	41	246.5	4.2	6.7	46.8	61.8
C3M1.4N8	52	76	229	6.6	7.8	62	80.2
C3M1.5N8	78	105	185.5	6.4	6.3	52.6	76.5

Note: FS denotes flexural strength; CS denotes compressive strength.

**Table 4:** Principal element content in different ratio groups.

Group	Ca (At%)	Si (At%)	Al (At%)	Na (At%)	Ca/Si	Al/Si	Na/Si
C0M1.2N8	7.91	10.60	4.65	6.08	0.75	0.44	0.57
C3M1.2N8	15.89	12.53	4.92	4.69	1.27	0.39	0.37
C10M1.2N8	6.67	8.30	4.61	4.45	0.80	0.56	0.54
C3M1.2N10	8.22	10.12	3.95	3.90	0.81	0.39	0.38
C3M1.4N8	6.98	8.92	3.51	5.77	0.78	0.39	0.64
C3M1.5N8	6.87	11.25	3.98	8.45	0.61	0.35	0.75

Note: The complete EDS data set includes all detected elements and is normalised to 100%. Table 4 lists only a selection of key elements used for mechanical interpretation.

**Table 5:** Carbon emissions and energy consumption of raw materials.

Raw Material	Embodied Carbon Emission (kgCO_2_/kg)	Embodied Energy Consumption (MJ/kg)	Reference
SAC	0.608	5.500	[63]
FA	0.008	0.100	[31,64,65]
GBFS	0.061	1.600	
CCR	0.375	1.48	
Sand	0.023	0.067	
Water glass	0.430	4.600	
NaOH	0.860	18.000	

**Table 6:** Environmental impact indicators of mortar (1 m^3^).

Group	Carbon Emission (kg/m^3^)	Energy Consumption (MJ/m^3^)	3d Strength (MPa)	Carbon Intensity (kgCO_2_eq/MPa)	Energy Intensity (MJ/MPa)
SAC	396.8	3339.5	42.5	9.34	78.6
C3M1.4N8	140.6	1716.9	62	2.27	27.7

## Data Availability

The original contributions presented in the study are included in the article.
